# Effects of Dietary Inclusion of a Proprietary Combination of *Quillaja saponaria* and *Yucca schidigera* on Intestinal Permeability and Immune Response in Broiler Chickens during a Coccidia Challenge

**DOI:** 10.3390/ani14121737

**Published:** 2024-06-08

**Authors:** Kari Saddoris-Clemons, Saheed Osho, Miriam Garcia, Brooke Humphrey

**Affiliations:** Phibro Animal Health Corporation, Teaneck, NJ 07666, USA

**Keywords:** broiler chickens, coccidia, cytokines, intestinal integrity, immune response

## Abstract

**Simple Summary:**

The implementation of antibiotic-free (ABF) and no-antibiotics-ever (NAE) poultry production has prompted interest in identifying viable options to reduce the need for medically important antibiotics (MIA) in animal feed. The Magni-Phi^®^ Ultra (MPU) nutritional specialty product is mainly composed of natural plant extracts (*Quillaja saponaria* and *Yucca schidigera*), which are known for their numerous benefits. By evaluating the effects of feeding MPU on the immune response and intestinal permeability of broilers during coccidia challenge at the peak and recovery phases of infection, this study found that intestinal integrity was enhanced and growth performance and immune response were improved when feeding MPU.

**Abstract:**

This study assessed the impact of Magni-Phi Ultra (MPU) inclusion on intestinal integrity and immunity in broiler chickens challenged with coccidia during peak and recovery phases. A total of 128 male Ross 708 broiler chicks were randomly allotted to one of four treatment groups (four chicks/cage). Treatments included an uninfected control (UUC); a coccidial challenge (CC) infected control (IUC); a CC fed salinomycin at 66 ppm (SAL); and a CC fed Magni-Phi Ultra at 0.11 g/kg of diet (MPU). At 16 days post-hatch, all birds in the CC groups were orally gavaged with a 3× dose of a live coccidia vaccine. At 5 dpi, the birds fed MPU and SAL showed decreased plasma FITC-d, oocyte shedding, and lesion scores and higher BWG compared to the IUC birds (*p* < 0.05). Jejunum *IL-17*, *IL-10*, and *IFN*-_ϒ_ mRNA expression was higher in the IUC compared to the UUC (*p* < 0.05) group at 5 dpi. At 12 dpi, the birds fed MPU or SAL had lower plasma FITC-d and jejunum *IFN*-_ϒ_ and *IL*-10 mRNA expression compared to the IUC birds (*p* < 0.05). This study indicates that MPU supports intestinal integrity and mucosal immune responses during the peak and recovery phases of infection, which may lead to improved health and performance.

## 1. Introduction

The implementation of antibiotic-free and no-antibiotic-ever poultry production has led to an increased prevalence of avian coccidiosis caused by *Eimeria* spp. infection. Coccidiosis is extremely costly to the poultry industry, with annual global losses estimated to be greater than USD 14.5 billion [1]. *Eimeria* spp. infections damage intestinal cells, compromise intestinal barrier function, cause inflammation, and induce oxidative stress. This damage often results in a reduction in nutrient absorption and ultimately worse growth performance and feed efficiency [2].

To help reduce the need for antibiotics, nutritional strategies such as plant extracts, essential oils, prebiotics, enzymes, minerals, probiotics, and organic acids have all been suggested as potential tools to help reduce coccidiosis-associated challenges [3,4,5,6]. These strategies are crucial in helping maintain poultry health and productivity. The phytogenic compounds, saponins, and polyphenols from *Quillaja saponaria* and *Yucca schidigera* have been shown to impact growth performance in broilers due to coccidiostat action and improvements in nutrient digestibility and intestinal permeability in both unchallenged and *Eimeria* challenge situations [2,7,8,9,10,11,12]. In addition, saponins have immunomodulatory effects [2,13]. Quillaja saponins stimulate mucosal immunity [13] and yucca saponins reduce pro-inflammatory effects [2] following immune challenges These compounds, due to their surfactant nature, disrupt protozoan cell membranes, leading to cell death, and offer antiprotozoal benefits [14]. Moreover, recent findings suggest they also increase ileal villus height and improve nutrient absorption [8], positively modulate the mRNA abundance of several tight junction proteins in the intestine [15], and alter the intestinal microflora in broiler chickens [16], pointing to their multifaceted role in avian health.

Previous studies both with and without yucca involving an *Eimeria* challenge have focused on the peak phase of *Eimeria* infection due to the high presence of oocysts and intestinal damage occurring during this period, which eventually leads to increased intestinal permeability [10,11,14,16,17,18]. However, little is known about the recovery phase (5 to 12 dpi) following an *Eimeria* challenge, when immune response and tissue repair are key. This phase also coincides with reduced oocyst shedding and clinical signs. Our study seeks to expand our understanding of the effects of feeding Magni-Phi Ultra (MPU) on broiler chickens infected with *Eimeria* by assessing its effects on growth performance, intestinal health, and immunity during both the peak and recovery phases following a coccidia infection. We hypothesized that the impact of coccidia infection would be reduced in broiler chickens with dietary supplementation of MPU during both the peak and recovery phases by improving intestinal health.

## 2. Materials and Methods

### 2.1. Animal Care

All animals were cared for adhering to both the guidelines outlined in the Phibro Animal Health Animal Care and Use Policy and the Guide for the Care and Use of Agricultural Animals in Research and Teaching [19].

### 2.2. Product

The product tested is a commercially available product, Magni-Phi Ultra (MPU; Phibro Animal Health Corp., Teaneck, NJ, USA). The product is a blend of triterpenoid saponins composed of a unique combination of two natural, plant-based products consisting of *Quillaja saponaria* and *Yucca schidigera* formulated in a proprietary ratio, where quillaja saponins are the major component. The product is formulated to contain 8% total saponins.

### 2.3. Bird Husbandry, Experimental Design, and Treatments

Ross 708 broiler chicks were purchased from a commercial hatchery (Welp Hatchery, Bancroft, IA, USA). Upon arrival, a total of 128 male chicks were housed across two colony brooders (Universal Brooder Box, GQF Manufacturing, Savannah, GA, USA). Throughout the experiment, the birds were exposed to a lighting schedule of 23L:1D. The first four days, chicks were offered water and a broiler starter feed ad libitum. Both brooder and chick room temperatures decreased as the animals aged. All birds were fed a corn–soybean meal mash basal diet formulated to meet or exceed the National Research Council (1994) nutrient requirements for broiler chickens (Table 1). The basal diet was devoid of coccidiostats and antibiotics. On d 6 post-hatch, the birds were individually tagged and weighed. The birds were then assigned randomly to 1 of 4 treatment groups utilizing a randomized complete block design. A total of 32 cages were utilized, with 8 cages per treatment and 4 birds per cage. The four treatments included T1, an uninfected, unchallenged control fed the basal diet (UUC); T2, a coccidosis challenge (CC) control fed the basal diet (IUC); T3, a CC fed the basal diet supplemented with salinomycin at 66 ppm (SAL-BioCox Granular 60, Huvapharma, Peachtree City, GA, USA); and T4, a CC fed the basal diet supplemented with Magni-Phi Ultra at 0.11 g/kg of diet (MPU). The birds were fed the experimental diets for 10 days prior to coccidia challenge.

### 2.4. Coccidia Infection, Lesion Scoring, and Oocyst Count

**Figure 1:**
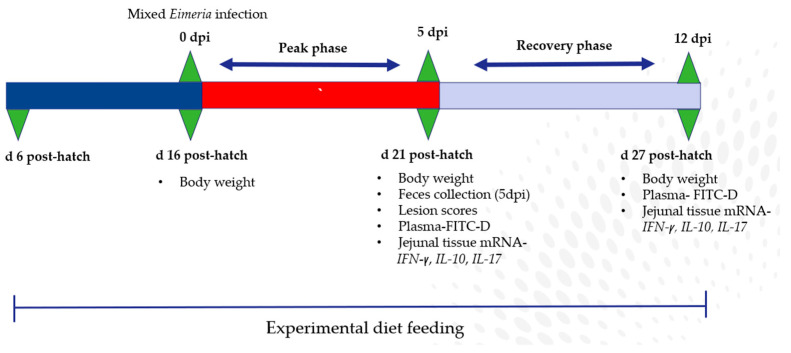
Schematic of study timeline and sampling. Study day 0 to challenge (d 16 post hatch) is represented by the blue bar, the peak challenge phase (d 16-d 21) is represented by the red bar, and the recovery phase (d 21-d 27) is represented by the light blue bar.

The birds in the IUC, SAL, and MPU groups were orally gavaged with a 3× dose of the live coccidia vaccine (Coccivac^®^ B52, Merck, Madison, NJ, USA), which was prepared from anti-coccidia sensitive strains from *Eimeria maxima*, *E. acervuline*, *E. tenella*, and *E. mivati* on d 16 post-hatch (0 dpi). Each vaccine bottle contains 10,000 doses of oocysts in an unspecified proportion of the included *Eimeria* species. The recommended dosage of the vaccine is 25 doses per kg of body weight (BW), meaning a 1 kg bird would require 25 doses, per the manufacturer’s instructions. At 16 days of age, a bird weighing 300 g would typically receive 7.5 doses of the vaccine. However, in order to induce a coccidia challenge, we administered 3× the recommended dosage, which was equivalent to 22.5 doses per bird. A 3× recommended dose was used to achieve a 10–15% reduction in BWG and a 2-fold increase in serum FITC-d during the peak challenge period. This vaccine dosage is similar in virulence to a live oocyst challenge model containing a mixture of 50,000 *E. acervuline* and 10,000 *E. tenella* oocysts utilized to induce a mild coccidiosis challenge [20]. The coccidia vaccine was gavaged in 1 mL at the appropriate concentration to the birds in the challenge groups (IUC, SAL, and MPU). The birds in the UUC group were orally gavaged with 1 mL of saline. Two birds per cage were randomly selected at 5 dpi and euthanized for scoring of duodenal lesions induced by coccidia infection. Duodenal lesions were scored by personnel blinded to treatment and were based on scores ranging from 0 (no gross lesion) to 4 (severe lesions) [21]. Excreta samples (~100 g) were collected at 5 dpi from each cage and stored in airtight plastic bags. Samples were homogenized and stored at 4 °C until assessed for oocyst counts. Oocyte counts were determined by dilution via a microscope utilizing a McMaster counting chamber [22] and are expressed as oocytes per gram of excreta. A description of the study sampling schematic is shown in Figure 1.

### 2.5. Growth Performance Measurements

The feed intake (FI) and BW of the birds were recorded per cage at 0, 5, and 12 dpi to evaluate body weight gain (BWG) and the feed conversion ratio (FCR) over the challenge period.

FCR = Body Weight Gain/Feed Intake

### 2.6. Gastrointestinal Permeability

Fluorescein isothiocyanate dextran (FITC-d; MW 4 kDa; Sigma-Aldrich Co., St. Louis, MO, USA) was administrated to evaluate intestinal permeability. Plasma FITC-d concentration was utilized as a marker of mucosal barrier dysfunction. At 5 and 12 dpi, two birds per cage were randomly selected and orally gavaged with 8.4 mg/kg BW of FITC-d (500 µL of FITC-d solution). The birds were euthanized by CO_2_ asphyxiation at 2 h following administration of the FITC-d. Blood (6 mL) was collected into EDTA vacutainer tubes (BD Company, Franklin Lakes, NJ, USA) and stored on ice. The blood was centrifuged at 2000× *g* at 4 °C for 10 min to separate plasma. A standard solution was created by diluting FITC-d with a pool of plasma from 5 unchallenged birds at both time points. FITC-d levels in both the standard solution and plasma samples were measured by florescence at an excitation wavelength of 485 nm and an emission wavelength of 530 nm utilizing a microplate reader (BioTek Instruments, Inc., Winooski, VT, USA).

### 2.7. Jejunum Collection and Real-Time Polymerase Chain Reaction Analysis

A mid-jejunum sample was collected at 5 and 12 dpi from 2 birds per cage for a quantitative real-time Polymerase Chain Reaction (PCR) (RT-qPCR) assay to quantify the relative mRNA abundance of interleukin-17 (IL-17), interleukin-10 (IL-10), and interferon γ (*IFN*-_ϒ_). Mid-jejunal samples were flushed with PBS, and a 1 cm section was removed and immediately submerged in an RNAlater^®^ stabilization solution (Thermo Fisher Scientific Inc., Waltham, MA, USA). Samples were held at room temperature for 1 h and then stored at −80 °C pending further analysis. RNA was extracted from the jejunal tissue utilizing a Tissue Purification Kit (NORGEN BioTek Corp, Thorold, ON, Canada), following the manufacturer’s recommendations. RNA concentration and purity were measured using a NanoDrop (ThermoFisher Scientific). The purity and concentrations of RNA samples were measured using a NanoDrop (ThermoFisher Scientific). The purity for all samples was between 1.80 and 2.03 for the 260/280 ratio. RNA was analyzed for the mRNA abundance of reference genes (GADPH and YWAHZ) and target genes (*IFN*-_ϒ_, *IL10*, and *IL17A*). Primers and TaqMan probes were obtained from Applied Biosystems (TaqMan^®^ Gene Expression Assays, Waltham, MA, USA). Primers were run in duplex pairs (I-GAPDH and *IL10-YWHAZ*) or singlets (*IFN*-_ϒ_). The lack of primer cross-hybridization was confirmed by verifying similar threshold cycle (Ct) values for the reactions in singlet and pairs.

For duplex reactions, primers were labelled with dyes with different fluorescence emission spectra (VIC or FAM). The RT-qPCR reaction volumes consisted of 5 µL of RNA (40 ng/µL), 9 (singlet) or 8 (duplex) µL of RNAse/DNase free water (ThermoFisher Scientific), 5 µL of Taqman Fast Virus 1-step Master Mix (cat no. 4444432 Applied Biosystems), and 1 µL of each primer. Each sample was set up in duplicate with specific primers and probes for chicken *IFN*-_ϒ_ (assay ID number Gg03348618_m1), chicken *IL10* (assay ID number Gg03358689_m1), chicken *IL17A* (assay ID number Gg03365522_m1), and the reference chick genes *GAPDH* (assay ID number Gg03346982_m1) and *YWHAZ* (assay ID number Gg03356701_m1) (Thermo Fisher Scientific). Samples for RT-qPCR reactions were run in duplicate using a CFX96 optics unit mounted on a C1000 touch base (BioRad, Hercules, CA, USA) in the fast-cycling mode. The PCR cycle parameters were an initial denaturing step at 95 °C for 20 s followed by 40 cycles of 95 °C for 3 s and 60 °C for 30 s. Target gene Ct-values were normalized to the geometric mean of the Ct-values of the two reference genes. The 2^−ΔΔCT^ method was used to calculate relative mRNA abundance, and the results are reported as fold-change relative to the UUC treatment group [23].

### 2.8. Statistical Analysis

A data analysis was conducted with the cage as the experimental unit using the GLIMMIX procedure of SAS 9.4 software (SAS Inst., Inc., Cary, NC, USA) with the random effect of block (initial BW) and the fixed effect of treatment. Data were tested for normal distribution in PROC UNIVARIATE using the Shapiro–Wilk test. In order to achieve normality, oocyst count data were natural log transformed. Differences between treatments were considered significantly different at *p* ≤ 0.05.

## 3. Results

### 3.1. Growth Performance

Broiler chicks were fed the dietary treatments for 10 days prior to the coccidia challenge. During this period (d 0–10), there was no difference in growth performance between treatment groups. Table 2 summarizes the effect of dietary treatments on growth performance during the coccidia challenge. In the peak phase (0–5 dpi), the BW, FCR, and BWG of the birds was significantly reduced (*p* < 0.05) in the IUC group compared to the UUC group. However, the birds fed SAL and MPU had similar BW, FCR, and BWG compared to the birds in UUC group. The birds in the SAL group consumed more (*p* < 0.05) feed than the other treatment groups and were heavier (*p* < 0.05) than the birds in the IUC and MPU groups. However, the SAL birds had similar BWG and FCRs when compared to the birds fed MPU. During the recovery phase (6–12 dpi), there were no differences in BWG and the FCR between treatment groups. However, feed intake was higher (*p* < 0.05) for the birds in the IUC group, followed by the other CC birds (SAL, MPU), with the UUC birds having the lowest feed intake. As for final BW, the birds fed MPU had similar BW when compared to the other groups, but the birds in the SAL group were heavier than the birds in the UUC and IUC groups.

### 3.2. Oocyte Count and Lesion Scores

Oocyte count and lesion scores are presented in Table 3. Dietary SAL and MPU supplementation significantly reduced (*p* < 0.05) oocyte shedding and duodenal lesion scores compared with the IUC group. The UUC birds remained free of coccidiosis infection, as indicated by the absence of duodenal lesions and oocyst shedding in that group.

### 3.3. Jejunal mRNA Expression Analysis

The differences between the treatment groups’ host immune responses were assessed by cytokine mRNA expression in the mid-jejunum of broiler chickens (Table 4). At the peak phase (5 dpi), the birds in the IUC group had significant upregulation (*p* < 0.05) of *IFN*-_ϒ_, *IL-17*, and *IL-10* compared to the other three groups, which had similar mRNA expression for all three measured cytokines. In the recovery phase, *IFN*-_ϒ_ and IL-10 remained upregulated (*p* < 0.05) in the IUC group compared to the other three groups. However, the birds fed SAL and MPU had similar *IL-10*, *IL-17*, and *IFN*-_ϒ_ mRNA expression when compared to the UUC group.

### 3.4. Intestinal Permeability

The alterations in intestinal permeability at 5 and 12 dpi are depicted in Table 5. The higher the concentration of FITC-d recovered in the plasma, the greater the intestinal permeability. Plasma FITC-d concentration was significantly higher (*p* < 0.05) in the birds in the IUC group compared to UUC birds at both 5 and 12 dpi. The birds fed SAL and MPU had significantly lower (*p* < 0.05) plasma FITC-d concentration at 5 dpi compared to the IUC birds. However, at 12 dpi, the birds fed MPU, but not SAL, had similar (*p* > 0.05) FITC-d concentrations when compared to the UUC group. As expected, 5 dpi had the most severe intestinal permeability across all treatment groups.

## 4. Discussion

### 4.1. Peak Phase

In the peak phase of coccidia infection, detrimental effects were observed on the broiler chickens’ health and performance, emphasizing the importance of exploring alternatives to enhance disease response without the use of antibiotics. As reported by previous studies, coccidia challenge has detrimental effects on intestinal integrity and nutrient absorption [20,24,25], which leads to altered expressions of immunoregulatory cytokines [26]. The results from this study confirm the negative impact of coccidia challenge in broiler chickens, even with the relatively lower challenge dosage utilized in this study. In addition, it investigated the effects of SAL or MPU on growth performance, intestinal permeability, and immune responses in coccidia-challenged broiler chickens. During the peak phase of coccidia infection (5 dpi), both the SAL and MPU treatments protected the broiler chickens from the negative impacts of the challenge, as evidenced by improved growth performance. The dietary inclusion of 500 mg/kg of a saponin-based product via an extract from *Yucca schidigera* effectively promotes growth in broilers infected with *Eimeria* [10]. However, the dietary inclusion of 250 or 500 mg/kg of a *Yucca schidigera*-based saponin source also had no impact on performance in broilers infected with *Eimeria* [2]. The saponin source and level of the product can vary greatly, and along with the severity of the coccidiosis challenge, impacts the efficacy. In the current study, the MPU treatment, which contained a blend of *Quillaja saponaria* and *Yucca schidigera*, was included at a much lower dosage of 110 mg/kg compared to other studies [2,10], indicating a higher potency of the *Quillaja Saponaria* compared to saponin products only from *Yucca schidigera* sources. To assess the severity of coccidia infection and its impact on the intestinal mucosa, we used intestinal lesion scores as an indicator. In this study, the birds in the IUC group exhibited a high oocyst count and duodenal lesion scores, which are indicative of an active coccidia infection. However, dietary supplementation with SAL or MPU decreased excreta oocyst counts and lesion scores. Both of these responses point to their effectiveness in reducing the replication or shedding of oocysts and protection against coccidia damage. The inclusion of *Quillaja saponaria* at 250 and 300 ppm reduced oocyst production (43% and 37%, respectively) and lesion scores following *Eimeria acervulina* and *Eimeria tenella* infection [14]. Yucca-based saponins also display coccidiostat actions, as measured by reduced oocyte production and protection against intestinal damage induced by the Eimeria in broilers [11]. Previous studies have demonstrated that saponins exhibit anti-protozoal properties through their interaction with cholesterol in protozoal cell membranes, leading to membrane damage and eventual cell lysis [27], and this effect has been observed in studies with both quillaja- and yucca-based saponin sources [10,14].

In the context of immune responses, cytokines play a crucial role in the immune system of animals [28]. T-cell-mediated immune response is an important factor in avian coccidiosis control. In this study, we chose to evaluate *IFN*-γ, a key cytokine that mediates the Th1 immune response; *IL-17*, a cytokine involved in the Th17-related immune response; and *IL-10*, a cytokine involved in the Treg-related immune response [29]. The Th17 immune response is reported to play a role in the early stages (2–4 dpi) of a coccidosis infection, while the Th1 and Treg immune response are involved in the later stages (6–8 dpi) of a coccidosis infection [29]. The peak phase (5 dpi) was characterized by a robust immune response in the IUC group relative to UUC, with marked upregulation of the cytokines *IL-17* (3×), *IL-10* (4.7×), and *IFN*-γ (3.9×), which is indicative of an active inflammatory and regulatory response to the coccidia infection. However, the SAL and MPU treatments modulated this acute response and maintained the expression of these cytokines at similar levels as the UUC group. This suggests that feeding these products may help to dampen the acute inflammatory response, potentially limiting the tissue damage associated with excessive inflammation. *Eimeria* infection, which damages the gut lining and disrupts barrier integrity, results in increased gut permeability and negative impacts on performance. This leakage, resulting from epithelial damage and tight junction disruption, can cause nutrient malabsorption and impaired immune responses, leading to secondary bacterial infections [26]. Our findings of reduced cytokine expression in the birds fed a saponin-based product during an *Eimeria* infection varies slightly from [2], who reported a reduction in *IL-1β* but no impact on *IFN*-γ or *IL-12β* in the duodenum at 7 dpi with birds fed 250 to 500 mg/kg of a *Yucca*-derived saponin. This indicates that a *Yucca*-based saponin has a lower impact on reducing pro-inflammatory cytokines involved in the Th1 immune response compared to saponin products containing *Quillaji*. *Quillaja* saponins have been suggested to act directly on T-helper cells of the mucosal immune system and induce T-cell and B-cell proliferation [30,31]. Saponin products containing both *Quillaja saponaria* and *Yucca schidigera* appear to have a greater immunomodulatory effect during an *Eimeria* infection compared to products with only *Yucca*-derived saponins. In the present study, elevated levels of plasma FITC-d following a mixed *Eimeria* spp. infection and duodenal lesion scores are indicative of the presence of intestinal damage. A previous *Eimeria* study also reported the highest intestinal permeability at 5 dpi [28]. This was coupled with negative impacts on performance during 0–5 dpi compared to the UUC group. Correspondingly, the reduced plasma levels of FITC-d in the SAL and MPU groups point to a protective effect on the intestinal barrier and a reduction in leakiness that is often a result of inflammation-induced damage. In summary, during the peak phase of coccidia infection, detrimental effects on the broiler chickens’ health and performance were evident, but the supplementation of the SAL and MPU treatments demonstrated promising protective effects. Feeding these products positively impacted growth performance, reduced lesion scores, modulated the acute immune response, and improved gut permeability, all of which contribute to mitigating the adverse effects of the coccidia challenge.

### 4.2. Recovery Phase

The current study continued to evaluate the effects of SAL and MPU treatments on broiler chickens as they progressed towards recovery from the coccidia challenge. In this phase, the birds in all feeding groups, including the IUC group, had similar growth and feed efficiencies, indicating recovery from the initial infection. However, the early protection provided by SAL and MPU may have allowed for a faster resolution of the inflammatory response and restoration of gut barrier integrity. By 12 dpi, the IUC group continued to exhibit higher expression of *IL-10* (2.6×) and *IFN*-γ (2.2×), but the degree of upregulation was about half of what was observed in the peak phase, indicating a shift from an acute response towards a resolving phase of the immune response. In all the CC birds, *IL-17*, a cytokine involved in the early immune response, had returned to levels similar to that of the UUC group. Our findings corroborate previous reports of increased mRNA expression of intestinal *IFN*-γ due to *Eimeria* infection in chickens during peak challenge (5–7 d post-infection), although the timing and magnitude of response vary considerably [32,33]. Resolution of the Th1, Th17, and Treg immune response and return of *IFN*-γ, *IL-17*, and *IL-10* cytokines to baseline levels was reported by 10 dpi in birds challenged with *E. tenella* sporulated oocytes at 18 d of age [29]. Differences in return to baseline of *IFN*-γ and *IL-10* in the current study may be due to the use of a mixed *Eimera* challenge model and the earlier age (16 d) at the time of challenge. By the recovery phase, *IL-10* and *IFN*-γ levels in the SAL and MPU groups were below that of the IUC group and similar to the UUC group. This may better enable tissue repair and the recovery of gut function, as shown by performance improvements in the recovery phase. *Yucca* and *Quillaja* saponins and polyphenols can inhibit inflammation in broilers raised under standard conditions, without any pathogen challenges, as reported by [16]. Another study also shows that *Yucca* bark and whole *Yucca* plant powder contain resveratrol, which is known for its anti-inflammatory activity [34,35]. Thus, whole-plant *Yucca* powder has powerful anti-inflammatory activity, mediated via inhibition of *NFkB* activation [27]. Dietary saponins from *Yucca schidigera* extracts included at levels as low as 100 mg/kg improve antioxidant capacity in the small intestine and increased gene expression of superoxide dismutase, glutathione peroxidase, and catylase [36]. This may help explain why a reduction of inflammation in broilers was observed during this coccidia challenge, but more research is needed, as limited research exists evaluating the immune response and antioxidant capacity of birds fed saponin-based products past the peak challenge phase.

To the best of our knowledge, publications on the effects of saponins on the intestinal permeability of broiler chickens during both the peak and recovery phases have not been well addressed in the literature. Our data suggest that MPU treatments are not only effective in reducing the impact of coccidia infection on gut barrier function during the peak phase but are also capable of supporting the recovery of gut integrity post-infection, as measured by plasma FITC-d levels. By 12 dpi, the IUC group continued to have elevated serum FITC-d levels (1.4×) compared to the UCC group. However, serum FITC-d levels of the MPU group were lower than the IUC group and had returned to levels similar to the UCC group, indicating recovery of gut barrier function.

Recovery of gut barrier function post-infection is critical, as it minimizes the risk of secondary complications associated with increased intestinal permeability, such as systemic inflammation or bacterial translocation. The observed alteration in intestinal permeability patterns may be linked to the ability of saponins to influence the permeability of intestinal cells through their interaction with cholesterol in mucosal cell membranes [37].

## 5. Conclusions

In conclusion, our study shows that the negative impact of a coccidia infection spans beyond the peak phase into the recovery phase, as evidenced by markers of inflammation (cytokines) and gut integrity (FITC-d levels) not yet reaching baseline (UUC) levels after 12 dpi. Also, our study provides new insights into the beneficial effects of MPU on intestinal permeability and immune responses during a coccidia challenge. We demonstrate that SAL and MPU treatments are effective not just in the acute phase but also in supporting recovery, highlighting their potential in improving intestinal integrity and growth performance. This contributes to the growing body of evidence supporting the use of saponins from both *Quillaja saponaria* and *Yucca schidigera* for their immune modulatory and gut health benefits, offering valuable alternatives to traditional antibiotic use.

## Figures and Tables

**Table 1:** Ingredients and nutrient composition of basal diet, g/kg as-fed basis.

Ingredients	g/kg
Corn	523.0
Soybean meal (47% CP)	380.0
Soybean oil	50.0
Calcium carbonate (38% Ca)	15.0
Dicalcium phosphate	15.0
Salt	4.0
Vitamin-mineral premix ^1^	5.4
_DL-_Methionine	3.8
_L-_Lysine.HCl	2.8
Threonine	1.0
Total	1000.0
Calculated Nutrients and Energy Content	
CP, g/kg	220.0
ME, kcal/kg	3134.0
Ca, g/kg	9.2
Ca:Tp	1.3

^1^ Supplied the following per kilogram of diet: vitamin A, 5484 IU; vitamin D_3_, 2643 IU; vitamin E, 11 IU; menadione sodium bisulfite, 4.38 mg; riboflavin, 5.49 mg; _D_-n-pantothenic acid, 11 mg; niacin, 44.1 mg; choline chloride, 771 mg; vitamin B_12_, 13.2 μg; biotin, 55.2 μg; thiamine mononitrate, 2.2 mg; folic acid, 990 μg; pyridoxine hydrochloride, 3.3 mg; I, 1.11 mg; Mn, 66.06 mg; Cu, 4.44 mg; Fe, 44.1 mg; Zn, 44.1 mg; Se, 300 μg.

**Table 2:** Growth performance, lesion scores, and oocyst shedding in broiler chickens fed diets supplemented with salinomycin (SAL) or Magni-Phi Ultra (MPU) during a coccidia challenge (dpi) ^1,2^.

	Uninfected	Coccidia Infection				
Item	UUC	IUC	SAL	MPU	SEM	*p*-Value
0–5 dpi (Peak Phase)						
Final body weight, g	570 ^ab^	515 ^c^	598 ^a^	563 ^b^	10.7	<0.01
Body weight gain, g/bird	264 ^a^	208 ^b^	287 ^a^	259 ^a^	11.8	<0.01
Feed intake, g/bird	301 ^b^	291 ^b^	332 ^a^	310 ^b^	7.4	0.04
Feed conversion ratio	1.15 ^b^	1.44 ^a^	1.17 ^b^	1.21 ^b^	0.07	0.03
6–12 dpi (Recovery Phase)						
Final body weight, g	1025 ^b^	1009 ^b^	1075 ^a^	1037 ^ab^	16.0	0.05
Body weight gain, g/bird	454	489	476	468	19.3	0.63
Feed intake, g/bird	612 ^c^	672 ^a^	640 ^b^	642 ^b^	9.2	<0.01
Feed conversion ratio	1.37	1.40	1.35	1.38	0.05	0.96

^a–c^ Means in the same row lacking a common superscript differ significantly (*p* < 0.05). ^1^ Data are least squares means of 8 cages per treatment. ^2^ Uninfected control (UUC) fed basal diet, coccidia challenge untreated control (IUC) fed basal diet, SAL (cocci challenge fed basal diet supplemented with salinomycin (66 ppm), and MPU (cocci challenge fed basal diet supplemented with Magni-Phi Ultra (0.11g/kg of diet)). On d 16 post-hatch (0 dpi), birds in the IUC, MPU, and SAL groups were orally gavaged with 3× the recommended dose of coccidia vaccine (Coccivac B52, Merck, Rahway, NJ), while birds in the UUC group were orally gavaged with 1 mL of saline.

**Table 3:** Lesion scores and oocyst shedding 5 dpi in broiler chickens fed diets supplemented with salinomycin (SAL) or Magni-Phi Ultra (MPU) during a coccidia challenge ^1,2^.

	Uninfected	Coccidia Infection				
Item	UUC	IUC	SAL	MPU	SEM	*p*-Value
Oocyst per gram of excreta (×10^2^)						
5 dpi	0 ^c^	81 ^a^	30 ^b^	28 ^b^	6.7	<0.01
Duodenum lesion scores						
5 dpi	0 ^c^	2.13 ^a^	1.13 ^b^	1.19 ^b^	0.17	<0.01

^a−c^ Means in the same row lacking a common superscript differ significantly (*p* < 0.05). ^1^ Data are least squares means of 8 cages per treatment. ^2^ Uninfected control (UUC) fed basal diet, coccidia challenge untreated control (IUC) fed basal diet, SAL (cocci challenge fed basal diet supplemented with salinomycin (66 ppm), and MPU (cocci challenge fed basal diet supplemented with Magni-Phi Ultra (0.11g/kg of diet)). On d 16 post-hatch (0 dpi), birds in the IUC, MPU, and SAL groups were orally gavaged with 3× the recommended dose of coccidia vaccine (Coccivac B52, Merck, Rahway, NJ, USA), while birds in the UUC group were orally gavaged with 1 mL of saline.

**Table 4:** Relative mRNA expression of cytokines in mid-jejunum tissue of broiler chicks fed diets supplemented with salinomycin (SAL) or Magni-Phi Ultra (MPU) during a coccidia challenge (dpi) ^1,2^.

	Uninfected	Coccidia Infection				
Item	UUC	IUC	SAL	MPU	SEM	*p*-Value
5 dpi (Peak phase)						
*IL-17*	1.00 ^b^	2.96 ^a^	1.64 ^b^	0.60 ^b^	0.02	0.04
*IL-10*	1.00 ^b^	4.72 ^a^	1.42 ^b^	2.00 ^b^	0.11	<0.01
*IFN*-_ϒ_	1.00 ^b^	3.89 ^a^	1.45 ^b^	1.56 ^b^	0.27	<0.01
12 dpi (Recovery Phase)						
* IL-17*	1.00	0.67	1.00	1.17	0.02	0.07
*IL-10*	1.00 ^b^	2.61 ^a^	1.64 ^b^	1.50 ^b^	0.24	0.04
*IFN*-_ϒ_	1.00 ^b^	2.19 ^a^	1.53 ^b^	1.22 ^b^	0.26	0.04

^a−b^Means in the same row lacking a common superscript differ significantly (*p* < 0.05). ^1^ Data are least squares means of 8 cages per treatment. *IL-17* = interleukin-17; *IL-10* = interleukin-10; interferon-gamma = *IFN*-_ϒ._^2^ Uninfected control (UUC) fed basal diet, coccidia challenge untreated control (IUC) fed basal diet, SAL (cocci challenge fed basal diet supplemented with salinomycin (66 ppm), and MPU (cocci challenge fed basal diet supplemented with Magni-Phi Ultra (0.11g/kg of diet)). On d 16 post-hatch (0 dpi), birds in the IUC, MPU, and SAL groups were orally gavaged with 3× the recommended dose of coccidia vaccine (Coccivac B52, Merck, Rahway, NJ, USA), while birds in the UUC group were orally gavaged with 1 mL of saline.

**Table 5:** Plasma fluorescein isothiocyanate dextran (FITC-d) concentration (ng/mL) of broiler chicks fed diets supplemented with salinomycin (SAL) or Magni-Phi Ultra (MPU) during a coccidia challenge (dpi) ^1,2^.

	Uninfected	Coccidia Infection				
Item	UUC	IUC	SAL	MPU	SEM	*p*-Value
5 dpi (Peak phase)						
FITC-d	46 ^c^	94 ^a^	70 ^b^	63 ^b^	4.79	<0.01
12 dpi (Recovery Phase)						
FITC-d	42 ^b^	59 ^a^	51 ^ab^	42 ^b^	5.59	<0.01

^a−c^ Means in the same row lacking a common superscript differ significantly (*p* < 0.05). ^1^ Data are least squares means of 8 cages per treatment. ^2^ Uninfected control (UUC) fed basal diet, coccidia challenge untreated control (IUC) fed basal diet, SAL (cocci challenge fed basal diet supplemented with salinomycin (66 ppm), and MPU (cocci challenge fed basal diet supplemented with Magni-Phi Ultra (0.11g/kg of diet)). On d 16 post-hatch (0 dpi), birds in the IUC, MPU, and SAL groups were orally gavaged with 3× the recommended dose of coccidia vaccine (Coccivac B52, Merck, Rahway, NJ, USA), while birds in the UUC group were orally gavaged with 1 mL of saline.

## Data Availability

The data presented in this study are available upon request from the corresponding author.
